# Physiological Reports begins a new manuscript category, the Methods article

**DOI:** 10.14814/phy2.15808

**Published:** 2023-08-31

**Authors:** Josephine C. Adams

**Affiliations:** ^1^ School of Biochemistry University of Bristol Bristol UK

Advancements in methods and technologies are key drivers of the frontiers of physiological research. With regard to the general mission of Physiological Reports to publish sound science, the Editors of Physiological Reports are pleased to announce the start of a new article type, the “Methods article.” Since its inception, Physiological Reports has published methods‐related articles on a great diversity of research topics. Examples from recent years have included uses of machine learning (Nolde et al., 2021), computational models for cardiomyocyte electromechanical applications (Forouzandehmehr et al., 2021); development of equations to assist customized prescriptions in clinical practice (Chen et al., 2023); noninvasive technologies to quantify bodily parameters or cardiovascular functions in humans or animals (Caterini et al., 2022, Naeve et al., 2019), and new quantification methods to apply to tissue samples (Schaubroeck et al., 2022). Because these articles were of necessity published within the original research article category, readers of the Journal could not easily sort methods articles as a single category. Also, a methods‐related topic could only be identified as such from the article title and/or a reading of the Abstract.

Our goals in starting a separate category for Methods articles are threefold. First, Physiological Reports will begin the publication of Methods articles as a route to disseminating knowledge that supports sound science through robust experimental methodologies. We welcome submission of Methods articles related to any research area of basic or translational physiology. Secondly, separating the Methods articles from Original Research articles will allow clarification of editorial and peer‐review expectations for submitted Methods manuscripts and will lead to a more consistent style for the content and presentation of Methods articles. These changes will benefit authors, readers, and peer‐reviewers. Thirdly, we aim to improve the visibility of the Methods papers to the global physiological research community by providing a general way to identify all future Methods articles through the article type.

Authors are invited to submit Methods articles directly for full peer‐review at Physiological Reports (https://physoc.onlinelibrary.wiley.com/journal/2051817x). We will also be pleased to consider Methods manuscripts transferred at the author's choice from the supporter journals; these manuscripts can be transferred with or without prior peer‐review. Manuscripts originally submitted to APS research journals as “Methods and Resources” articles, to The Journal of Physiology as “Techniques for Physiology” or to Experimental Physiology as techniques‐related “Short Communications” will be well‐aligned to the Physiological Reports “Methods article” category. In starting up Methods articles at Physiological Reports, we also anticipate to publish occasional Review articles on methods‐related topics; for example, to discuss the underlying principles of selected methods or technologies; to compare/contrast related methods and their strengths and limitations, or to provide a synthesis on methods relevant to the study of a particular organ, tissue, or molecule.

As for all article types at Physiological Reports, authors may make the initial submission in the manuscript format of their choice. With regard to the content of Methods articles, we have set out several general requirements and guidelines to distinguish the Methods articles from Research articles. The aims are to ensure that the articles will be of high value to readers for their own research and to provide consistency in article structure and general quality. Full details of the guidelines can be found at (https://physoc.onlinelibrary.wiley.com/hub/journal/2051817x/about/author‐guidelines/methods_in_physiology). In brief, we ask for a concise Introduction, with a short overview of the context and rationale for the development of the method, followed by a Methods section that is sufficiently detailed that others can follow or adapt the method. For laboratory‐based Methods, catalogue numbers or Research Resource Identifiers (RRID) (https://scicrunch.org/resources) should be given for essential reagents. The Results section should include the controls and validations used to set up the method or computational model, along with an initial demonstration of an application of the method in a physiological context. A short section in the Discussion should comment on the strengths and limitations of the method.

In addition, as a Gold Open Access journal, Physiological Reports supports open data sharing, such as supported by the Center for Open Science (https://www.cos.io). For example, the referencing of step‐by‐step, Open protocols, registered methods, or video demonstrations of a complex laboratory procedure can supplement the value of the information provided in the Methods Article itself. Extensive information on data sharing and the choice of an appropriate recognized repository or database is provided by the publisher (https://authorservices.wiley.com/author‐resources/Journal‐Authors/open‐access/data‐sharing‐citation/data‐sharing‐policy.html).

The Editors look forward to the growth and development of Methods articles at Physiological Reports as a valuable new resource for physiological research. Pre‐submission enquiries or suggestions for topics for methods‐related Reviews are welcome and can be sent to the Editor‐in‐Chief at jo.adams@bristol.ac.uk.

## FUNDING INFORMATION

No funding was associated with the preparation of this editorial.

## CONFLICT OF INTEREST STATEMENT

There are no conflicts of interest or other ethical issues to declare.

## Data Availability

There are no original data associated with this editorial.
